# Chromatin Conformation in Development and Disease

**DOI:** 10.3389/fcell.2021.723859

**Published:** 2021-08-04

**Authors:** Ilias Boltsis, Frank Grosveld, Guillaume Giraud, Petros Kolovos

**Affiliations:** ^1^Department of Cell Biology, Erasmus Medical Centre, Rotterdam, Netherlands; ^2^Cancer Research Center of Lyon – INSERM U1052, Lyon, France; ^3^Department of Molecular Biology and Genetics, Democritus University of Thrace, Alexandroupolis, Greece

**Keywords:** chromatin conformation, TAD, development, differentiation, disease, cancer, gene regulation, regulatory element

## Abstract

Chromatin domains and loops are important elements of chromatin structure and dynamics, but much remains to be learned about their exact biological role and nature. Topological associated domains and functional loops are key to gene expression and hold the answer to many questions regarding developmental decisions and diseases. Here, we discuss new findings, which have linked chromatin conformation with development, differentiation and diseases and hypothesized on various models while integrating all recent findings on how chromatin architecture affects gene expression during development, evolution and disease.

## Introduction

All eukaryotic species share the ability to reproduce and transmit their genetic information to their offspring. Mammals originate from single cells, with all the hereditary information stored in the DNA. The 2 meters of chromatin, consisting of DNA plus associated proteins must be compacted to fit in a nucleus with a diameter that varies between 2 and 10 μm.

The chromatin fiber is a highly dynamic polymer undergoing cycles of de-compaction and re-compaction during the cell cycle and proliferation/differentiation of the cells (Woodcock and Ghosh, 2010). Compaction impacts on chromatin accessibility to transcription factors (TF) and RNA polymerases (RNAPs) and is one of the parameters that fine-tunes the regulation of gene transcription. Thus, different cell fates require a different three-dimensional genome architecture that is closely related to gene expression and cellular function (Dixon et al., 2015). The nuclear genome appears to be organized non-randomly, through a variety of chromatin loops and rosettes and suggests that transcription is also architecturally organized (Lanctôt et al., 2007). Recent data suggest that alterations in chromatin architecture could be causal in diseases and cancer (Spielmann et al., 2018). Here, we describe recent findings about the relation between chromatin conformation and gene regulation in development and diseases and propose a model for chromatin architecture and the formation of loops during development.

## High-Order Chromatin Structures

### Chromosomal Territories

**FIGURE 1:**
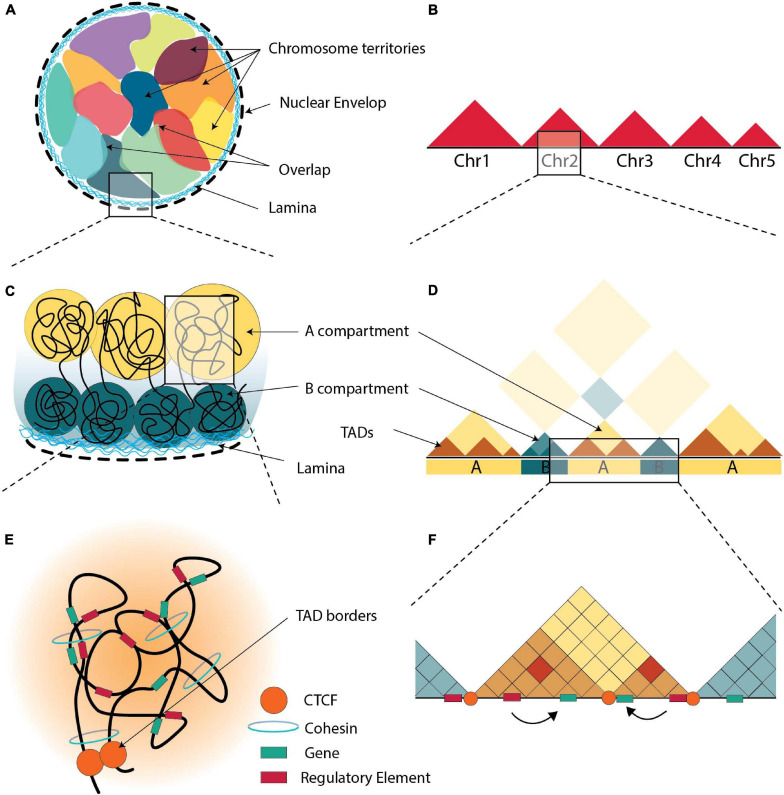
The 3D organization of chromatin. **(A)** Schematic representation of the arrangement of chromosomes in nucleus, all chromosomes are in contact with the nuclear envelop i.e., the nuclear lamina. Each chromosome resides in its territory (multicolor areas), but there are areas of overlapping. **(B)** Schematic illustration of Hi-C maps at the genomic scale of chromosomes. When compared to inter-chromosomal connections, intra-chromosomal interactions are found to be more prevalent. **(C)** Chromatin is organized in “**A**” (yellow) and “**B**” (green) compartments, with “**B**” compartments being at the nuclear lamina. **(D)** Schematic illustration of Hi-C maps at the compartmental scale, where distal chromatin contacts generate a distinctive plaid pattern with A and B compartments. **(E)** TADs are formed via loop extrusion, and architectural proteins are found near the TAD boundaries. Within each TAD, cohesin-mediated loops contribute in chromatin folding. **(F)** Schematic illustration of Hi-C maps at the sub-megabase scale, TADs appear as interaction-rich triangles separated by TAD borders. Through loops, enhancers are brought into closer to the promoters that they control.

Although the sequence of many genomes has been elucidated, the study of its 3D organization is subject to increasing endeavors using a variety of techniques, most prominent of which are 3C related technologies and high-resolution microscopy. Chromatin is divided into a dark and a light electron-dense region, representing heterochromatin and euchromatin, respectively and gene activity is related to the position of the genes in the 3D chromatin architecture (Shachar and Misteli, 2017). The sub-nuclear space occupied by a chromosome is called ‘’chromosomal territory” (CT) (Figures 1A,B; Cremer and Cremer, 2001; Dixon et al., 2016). On a smaller scale, the genome contains two levels of topological organization, one at a megabase level (A/B compartments) and one at sub-megabase level (topologically associated domains, TADs) (Dixon et al., 2016).

While chromosomes generally reside in distinct territories, CTs sometimes overlap (Branco and Pombo, 2006). These overlapping areas have been suggested to have a functional role in gene regulation suggesting that co-transcription of multi-gene complexes is hierarchical and may require intra and inter-chromosomal interactions (Fanucchi et al., 2013).

### Chromosomal Compartmentalization and Its Dynamic Nature

Inside CTs, chromosomes are thought to be divided in two compartments. The large multi-Mb euchromatic A-compartments occupy the internal regions of the nucleus with generally actively transcribed genes, while the heterochromatic B-compartments occupy the periphery of nuclei containing inactive genes (Figure 1C; Lieberman-Aiden et al., 2009; Denker and de Laat, 2016; Szabo et al., 2019). However, in some cases, the positions of A and B compartments inside the nucleus are inverted, indicating the dynamic relationship between heterochromatin and euchromatin (Falk et al., 2019).

DNA regions interact more frequently with regions in the same compartment rather than with regions in other compartments (Figure 1D; Robson et al., 2019). Every cell type expresses a different set of genes and therefore the content of A/B compartments is cell-type specific. A/B compartments are highly dynamic and change according to the requirements of the cell (Corces et al., 2016; Javierre et al., 2016; Schmitt et al., 2016; Azagra et al., 2020), and the availability of transcription factors and chromatin-modifying enzymes (Therizols et al., 2014; van Steensel and Belmont, 2017), although ∼40% has little variability among different human tissues and cell types (Schmitt et al., 2016). Various studies suggest that genes reposition from the periphery to the nuclear interior and vice versa during cell differentiation to activate or repress genes (van Steensel and Furlong, 2019). Such a compartment switch from B to A is observed during T-cell differentiation, where *BCL11B* is activated and the entire locus moves from the periphery to the center of the nucleus (Isoda et al., 2017). Another example is the rearrangement of the *Igh* locus in mice from the periphery to the center of the nucleus during B cell maturation (Kosak et al., 2002). The opposite switch also occurs, e.g., during neuroblast formation in D. melanogaster where the hunchback (*hb*) gene moves to the nuclear lamina (Kohwi et al., 2013). Interestingly, 36% of A/B compartments of human genome switched from an open to closed state and/or *vice versa* during differentiation, while maintaining their TAD boundaries (Dixon et al., 2015). The number of B compartments increases during differentiation from embryonic stem cells to differentiated cells (Xie et al., 2013). Nevertheless, the expression patterns of the majority of genes did not change (Dixon et al., 2015). Thus, compartmentalization seems to be dependent on the levels of transcription in a genomic region, and not the expression patterns of each gene (Zheng and Xie, 2019). Recently, an intermediate compartment termed ‘I’ was identified in maturing B-cells, which contains mainly poised promoters and Polycomb-repressed chromatin states (Vilarrasa-Blasi et al., 2021).

### Nature, Topology and Role of Topologically Associated Domains

The second sub-megabase level of topological organization comprises compartments which are organized in self-associating domains and are divided by linker regions. These compartments are called “topologically associated domains” (TADs) (Figure 1E; Dixon et al., 2012). This organization facilitates physical contacts between genes and their regulatory elements (Nora et al., 2012; Sexton et al., 2012) and range between 0.2 to 1 Mbp (Dixon et al., 2012; Nora et al., 2012; Sexton et al., 2012). Contacts between regulatory elements are more frequent inside a particular TAD rather than between two different TADs (Figure 1F; Nora et al., 2012).

TADs are highly conserved upon stem cell differentiation, reprogramming, stimulation and in different cell types (Bonev and Cavalli, 2016; Andrey and Mundlos, 2017; Flyamer et al., 2017; Sauerwald and Kingsford, 2018; Zheng and Xie, 2019). Many differentiated cell types contain hundreds of TADs similar to those of human ESCs (Dixon et al., 2015; Schmitt et al., 2016). Thus, TADs are regarded as the basic unit of the folded genome and are considered as structural elements of chromosomal organization (Cremer and Cremer, 2001; Dekker and Heard, 2015; Sexton and Cavalli, 2015). TADs may also not appear as stable structures in single cells, but rather as a mix of chromatin conformations present in a population of cells (Nora et al., 2012; Flyamer et al., 2017; Zheng and Xie, 2019). A multiplexed, super-resolution imaging method identified TAD-like structures in single cells, although these were not stable (Bintu et al., 2018). Similar observations were made even between individual alleles (Finn et al., 2019). Interestingly, a number of studies have indicated that TADs could also be conserved between species (Rudan et al., 2015; Harmston et al., 2017; Krefting et al., 2018), while others come to the conclusion that that TADs certainly have some functional conservation but that specific TAD structures and their location may not be conserved (Eres and Gilad, 2021). This difference in conclusions suggests that the observed various sorts of conservation could be the result of study designs and/or different analytical choices.

As discussed in recent reviews (Sexton and Cavalli, 2015; van Steensel and Furlong, 2019), TADs could affect gene expression in various ways. TADs play an important role in regulation of gene expression by either acting as barriers or by facilitating or preventing loop interactions, because two points (regulatory elements) tethered on a string interact more frequently (Figure 1F; Dillon et al., 1997; Lieberman-Aiden et al., 2009; Symmons et al., 2014; Bonev and Cavalli, 2016; Bompadre and Andrey, 2019; Robson et al., 2019; Schoenfelder and Fraser, 2019; Sun et al., 2019). Importantly TADs appear to be lost during mitosis and cell division and to be re-established only after the formation of cis regulatory interactions, which suggests they are not driving but rather maintaining genome structure (Giorgetti et al., 2013; Naumova et al., 2013; Espinola et al., 2021). Disruption of TAD boundaries can nevertheless alter promoter-enhancer interactions by allowing new or preventing normal interactions (Lupiáñez et al., 2015; Flavahan et al., 2016). While TAD boundaries are generally conserved across cell types, a small fraction exhibit cell-type specificity with changes observed within boundaries during differentiation (Dixon et al., 2012, 2015; Zheng and Xie, 2019). It is worth mentioning here, that the location of boundaries in single-cells varies from cell-to-cell but is always located close to CTCF and cohesin binding sites. Stable TAD boundaries could only be observed in population averaging studies (Bintu et al., 2018). Changing the enhancer-promoter distance within a TAD has little effect on the gene’s expression level (Symmons et al., 2016), unless multiple genes compete for interactions with the enhancer (Dillon et al., 1997). However, inversions, that disrupt the TAD structure alters expression levels (Lupiáñez et al., 2016; Symmons et al., 2016; Robson et al., 2019). Potentially TAD boundaries could be as barriers to prevent the spread of heterochromatin to active regions (and *vice versa*) and/or the spread of proteins tracking on the chromatin (Austenaa et al., 2015; Narendra et al., 2015). One of the main roles of TADs is to provide an insulation for the enhancer-promoter interactions and contain them within the TAD (Dixon et al., 2012; Rao et al., 2014; Zhan et al., 2017; Gong et al., 2018), although there are cases where enhancer-promoter interactions cross over the TAD boundaries, such as in human hematopoietic cells (Javierre et al., 2016) and between Polycomb-bound regions in mouse ESCs (Schoenfelder et al., 2015b; Bonev et al., 2017; Schoenfelder and Fraser, 2019).

The position of TADs in the nucleus relative to each other, or to the nuclear periphery or substructures is under intense investigation. Localization has been proposed to influence gene expression, such as the observation that TADs containing repressed genes at a particular developmental stage are localized at the nuclear lamina (Guelen et al., 2008). Some heterochromatic TADs correspond to lamina associated domains (LADs) or parts of the genome with repressive histone marks (Nora et al., 2012). This agrees with studies suggesting that LADs are poor in genes and that their transcription is suppressed (Lanctôt et al., 2007; Guelen et al., 2008). LAD and heterochromatic TAD regions overlap, albeit incompletely (van Steensel and Belmont, 2017). Euchromatic TADs are transcriptionally active and correspond to regions with active histone marks (Dixon et al., 2012; Nora et al., 2012; Sexton et al., 2012). Erasing the histone modifications did not affect TAD conformation, possibly because these histone marks are formed in pre-existing TADs (Nora et al., 2012; Dekker and Heard, 2015). LADs and euchromatic TADs are clearly separated by defined borders of CTCF or active promoters (Guelen et al., 2008). Interestingly, in *D. melanogaster*, most of the TAD borders correspond to regions of active promoters rather than CTCF-binding sites (Ramírez et al., 2018). Similar observations were also made in mESCs (Bonev et al., 2017).

### The Important Regulators of Genome Organization

Several key proteins are involved in the establishment of chromatin loops and domains with CTCF and cohesin being among the most studied (Dixon et al., 2012; Rao et al., 2014; Fudenberg et al., 2016; Kim et al., 2019). Proper chromatin interactions require convergent pairs of CTCF bound regions, marking the boundary sites of a TAD (Phillips-Cremins et al., 2013; Zuin et al., 2014; de Wit et al., 2015; Guo et al., 2015; Jia et al., 2020). Inverting or deleting the CTCF sites could affect chromatin conformation, leading to an increase of inter-domain contacts and a decrease of intra-domain contacts (Dixon et al., 2012; de Wit et al., 2015; Hanssen et al., 2017). CTCF is enriched in TAD boundaries (Dixon et al., 2012; Nora et al., 2012), although its presence is not limited to boundary sites (Zuin et al., 2014). It is also important to note that while CTCF loops define a subset of TADs (Dixon et al., 2012; Nora et al., 2012; Sexton et al., 2012), not all TADs are surrounded by CTCF sites (Rao et al., 2014). Importantly, CTCF disruption changes TAD structure (de Wit et al., 2015; Guo et al., 2015; Narendra et al., 2015; Sanborn et al., 2015; Nora et al., 2017), while TADs dramatically disappear after depletion of cohesion and compartmentalization is increased (Haarhuis et al., 2017; Rao et al., 2017; Schwarzer et al., 2017; Wutz et al., 2017). Interestingly, these results were corroborated by polymer simulations (Nuebler et al., 2018). Moreover, CTCF interacts with the cohesin complex, which was proposed to organize the genome based on loop extrusion (Fudenberg et al., 2016). It should be noted though that it has not been shown yet that cohesin loops are formed through extrusion *in vivo*. The extrusion mechanism of cohesin is an asymmetric process, which would have certain implications on gene expression. Interestingly recent data indicate that cis regulatory loops are already formed after mitosis before TADs are formed (Espinola et al., 2021).

An example of a topological organization of a locus that could be explained based on the loop extrusion model is that of the α-globin locus (Brown et al., 2018). The self-interacting domain is not present in mES cells, but is formed in differentiating erythroblasts with no apparent change in the binding of CTCF (Brown et al., 2018). Upon perturbations that abolish the expression of α-globin, the domain conformation was unaffected, although interactions within the domain were significantly altered. The convergent pair of CTCF bound regions do not appear as a unique contact, but a broader area of tissue specific contacts was observed around the CTCF borders (Brown et al., 2018). Other mechanisms such as transcription could also lead to loop extrusion. Different cohesin complexes with different subunits (SA1, SA2) seem to act in a different manner mediating different aspects of DNA conformation. SA1-containing complexes promote TAD formation/stabilization while SA2-containing complexes mediate intra-TAD enhancer-promoter contacts (Kojic et al., 2018), suggesting that transcription and transcription factors are important in the formation of those domains. Loop extrusion is also supported by computational modeling (Fudenberg et al., 2016) and also by perturbation assays of important factors of 3D chromatin conformation, such as CTCF and cohesin (Sofueva et al., 2013; Haarhuis et al., 2017; Nora et al., 2017; Rao et al., 2017; Schwarzer et al., 2017; Wutz et al., 2017; Schoenfelder and Fraser, 2019; Thiecke et al., 2020).

DNA is thought to asymmetrically slide through the cohesin ring until it reaches a CTCF site where cohesin is stalled to stabilize the loop (Nuebler et al., 2018). It has been proposed that loop extrusion initiates where cohesin is loaded on DNA through the NIPBL protein. Experiments *in vitro* have shown that human cohesin–NIPBL complexes extrude loops in an ATP-dependent manner (Kim et al., 2019; Golfier et al., 2020).

The removal of NIPBL highlighted two different mechanisms for the genome organization. One is independent of cohesin and organizes the genome into fine-scale compartments (compartmentalization), while the other is dependent on cohesin and contributes to the formation of TADs (Schwarzer et al., 2017; Thiecke et al., 2020). In fact, depletion of CTCF had little effect on A/B compartments, while depletion of cohesin even strengthens it (Nora et al., 2017; Rao et al., 2017; Schwarzer et al., 2017; Wutz et al., 2017; Cremer et al., 2020). This is further supported from experiments where RAD21, a subunit of cohesin complex, was degraded, which disrupted all CTCF loops indicating that CTCF alone cannot stabilize the loops. After restoring RAD21, the majority of CTCF loops appeared within 40 minutes (Fudenberg et al., 2016; Hansen et al., 2017). These findings contradict the hierarchical organization model that suggests that TADs are the compartmental building blocks and suggests that the loop extrusion may change compartmental features (Nuebler et al., 2018). The unloading of cohesin is ensured by other proteins such as WAPL and PDS5 (Wutz et al., 2017). Lack of WAPL contributes to loop collision, with an increase of interactions between distal CTCF sites due to an incremental aggregation of loop domain anchors, and thus, creating a “cohesin traffic jam” (Allahyar et al., 2018). Whether cohesin is “fixed” at CTCF sites remains elusive. It was shown that CTCF and WAPL bind to the same cohesin pocket, with CTCF stabilizing cohesin at TAD boundaries and thus blocking WAPL action (Li et al., 2020). The binding signals at CTCF binding sites are higher than at other position in the genome (Sanborn et al., 2015), but the low general background signal could indicate that cohesin is loaded and extruding continuously and only has a longer dwell time at CTCF sites (Fudenberg et al., 2016, 2017).

CTCF mediated RNA interactions are essential for the proper genome organization (Saldaña-Meyer et al., 2019). Furthermore, many long non-coding RNAs (lncRNAs) have been found to interact with chromatin (Chu et al., 2011; Simon et al., 2011; Engreitz et al., 2013; Li et al., 2017), suggesting that lncRNAs are involved in structural organization of the genome, like Xist and Firre. During X chromosome inactivation, the lncRNA Xist controls the conformation of the inactive X chromosome (Splinter et al., 2011; Engreitz et al., 2013; Chen et al., 2016), while Firre facilitates the colocalization of genomic regions from different chromosomes (Yang et al., 2015). Moreover, T-cell fate is determined by the lncRNA ThymoD and its role to promote promoter-enhancer interactions (Isoda et al., 2017). Nonetheless, further research is needed in order to conclude, whether lncRNAs play a role in structural organization of the genome.

### Higher/Lower Levels of Genome Organization

**FIGURE 2:**
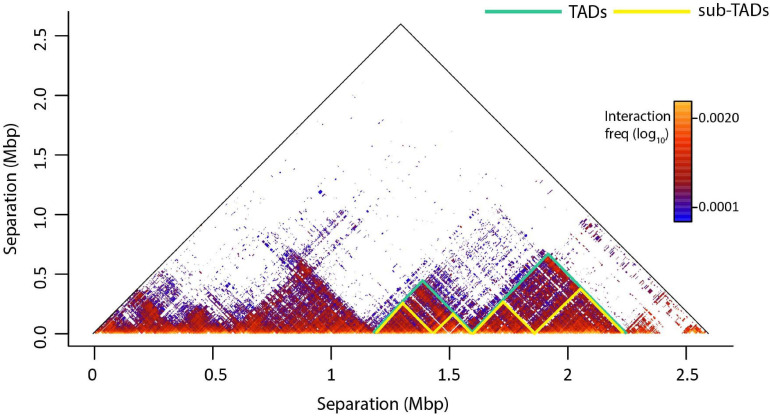
An example of genome architecture: TADs and sub-TADs. T2C interaction frequencies are displayed as a two-dimensional heatmap, where intra-TAD contacts (in fact proximities) are more frequent than inter-TAD contacts. TADs confine cis-regulatory elements and target gene promoters in space like two elements tethered on a string. This facilitates regulatory interactions within the TAD and prevents unwanted regulatory activity across TAD regions. Sub-TADs and TADs are depicted with yellow and green lines, respectively.

Topologically associated domains are further divided into smaller organizations, the sub-TADs which have a median size of ∼185 Kbp and are characterized by higher interaction frequencies (Figure 2; Rao et al., 2014; Rowley et al., 2017). Sub-TADs should not to be confused with the compartmental domains, which are not formed by CTCF loops but the segregation of A/B compartments (Rowley et al., 2017; Rowley and Corces, 2018). Compartmental domains have been proposed as a model for the organization of chromatin, with architectural proteins and TAD boundaries contributing in the fine-tuning of the transcriptome or regulating a subset of the genes (Stadhouders et al., 2019). On the other hand, a sub-TAD could contain one (or more) gene(s) with its/their regulatory elements, leading to their transcriptional activation or repression (Phillips-Cremins et al., 2013; Rao et al., 2014; Symmons et al., 2014; Bonev et al., 2017). TADs may also contact each other on a higher scale, forming meta-TADs in which inter-TAD interactions are favored (Fraser et al., 2015). sub-TADs and/or meta-TADs exhibit more tissue specific interaction patterns than the tissue invariant TADs (Dixon et al., 2016; Andrey and Mundlos, 2017).

Other levels of chromatin organization are loop domains and insulated neighborhoods (Rao et al., 2014; Hnisz et al., 2016a; Andrey and Mundlos, 2017). Loop domains are regions with enriched interactions marked by a loop at their border (Rao et al., 2014). A loop domain can represent a whole TAD, but also only a part of it. The current mainstream hierarchical model of chromatin organization promotes, that compartments contain several TADs and subsequently contain several sub-TADs, suggesting that if TADs are the building blocks of the genome, sub-TADs would be the cement holding them together (Bonev and Cavalli, 2016). Insulated neighborhoods are genomic domains, encompassing at least one gene and forming chromatin loops, which are sealed by a CTCF homodimer and co-bound with cohesin (Hnisz et al., 2016a).

### Limitations of Methods Unveiling TADs

At present, genome-wide identification of both TADs and sub-TADs relies on the resolution of 3C related technologies and at least 22 different computational methods, contributing to the argument that TADs may not be a “discrete” level of organization of the genome (Fudenberg and Mirny, 2012; Rao et al., 2014; Xu et al., 2020). Nevertheless, genes within the same TAD show similar expression patterns across multiple types of cells and tissues, a trait that is substantially lower at other levels of organizational. This observation favors the role of TADs as a functional level of organization where gene regulation takes place. It is however worrying that different experimental methods result in different estimates of TAD size and numbers (Zufferey et al., 2018), possibly due to the low coverage of the 3C related technologies (Xu et al., 2020) and the different models that each algorithm employs. Adding to this, in single-cell Hi-C experiments, TADs are not reproducibly detected at individual loci, but may be ”reassembled” when the individual maps are combined to create a whole population (bulk) experiment (Flyamer et al., 2017). The inherent problem here is that each fragment has only two ends and thus, it could be ligated with four only other fragments. Moreover, contacts are dynamic, created and lost all the time, with TAD borders seeing each other more frequently, strengthens the notion that a TAD is only visible when many cells are analyzed. Thus, the need of improved chromatin conformation capture techniques with increased resolution and coverage as well as algorithms identifying consistently TADs is of prime importance.

## Enhancer-Promoter Contacts as the Driving Force of Transcription

### Looping (*de novo* Contacts)

**FIGURE 3:**
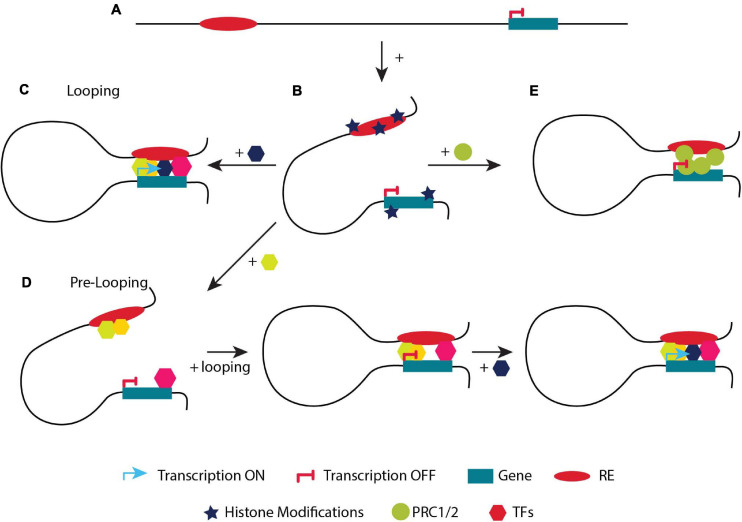
A simplified example of the two models regulating transcription. **(A)** Linear distance of a regulatory element (RE) and a gene. **(B)** The addition of active or repressive histone marks will determine if the gene will be placed in an A or B compartment. **(C)** Looping model. Describes the classic model of activation of a gene upon looping. An enhancer is not in close proximity with its target gene and therefore it is not transcribed. Upon the binding of different TFs, looping of the enhancer to the target gene takes place and the gene is expressed. **(D)** Pre-looping model. The gene is in close proximity (looped) with the enhancer but not actively transcribed. However, recruitment of an additional (crucial) TF to the enhancer (depicted in a dark blue hexagon) initiates transcriptional activation of the promoter while preserving their close proximity. **(E)** The gene is in close proximity (looped) with the enhancer but not actively transcribed. The gene is placed in a **(B)** compartment, bound by the PCR complex.

Gene transcription is tightly regulated by regulatory elements (enhancers, insulators, silencers), which can be located at various distances from their cognate gene(s) on the linear DNA strand (Figure 3A; Kolovos et al., 2012; Schoenfelder et al., 2015a; Sun et al., 2019). In order to carry out their function, regulatory elements have to be in close proximity to their target gene(s) (Stadhouders et al., 2019). ‘Loops’ between enhancers and promoters usually result in local interactions, as opposed to CTCF-mediated long-range chromatin loops (TADs), which could facilitate enhancer-promoter interactions either by bringing them closer or by segregating the genome according to its chromatin state (Figure 3B; Zheng and Xie, 2019). Recently it was shown that TFs (e.g., YY1 and LDB1), ncRNAs, the Mediator complex, p300 acetyltransferase and the cohesin complex proteins play key roles in the stabilization of chromatin looping or transcription factories (Kagey et al., 2010; Stadhouders et al., 2012; Phillips-Cremins et al., 2013; Fang et al., 2014; Zuin et al., 2014; Schoenfelder et al., 2015a; Boija et al., 2018; Cho et al., 2018; Spielmann et al., 2018; Peñalosa-Ruiz et al., 2019). The function of cohesin varies between various promoter-enhancer interactions. Some promoter–enhancer interactions could also be established only by transcription factors without the involvement of cohesin (Rubin et al., 2017). Four models have been proposed to explain how promoters and enhancers may regulate gene expression with the looping and the transcription factory model being the most prominent (Kolovos et al., 2012; Papantonis and Cook, 2013). Notably, the general notion of the looping model is that an enhancer is in close proximity to its target promoter(s) leading to gene activation, while the gene is silenced when the enhancer and promoter are not in close proximity.

Gene regulation from distal regulatory elements through local looping is now a commonly accepted concept (Lupiáñez et al., 2015; Flavahan et al., 2016; Hnisz et al., 2016a; Bonev et al., 2017; Stadhouders et al., 2018). Before the development of chromosome conformation capture technologies, which are essentially biochemical techniques, there was already strong evidence from biochemical and genetic type experiments that loop formation mediates transcription in both prokaryotic and eukaryotic systems. That was depicted *in vitro* with the lac repressor system (Hochschild and Ptashne, 1986). In eukaryotic systems, *in vitro* assays using a plasmid suggested that an enhancer and a gene could be separated by a protein bridge invoking looping (Müeller-Storm et al., 1989). Strong evidence in eukaryotes, with genes in the normal genome environment, was obtained at the β*-globin* locus after discovery of the Locus Control Region (LCR, (now called super-enhancers), which is located 70 kb upstream of the β*-globin* gene(s). Changing the distance or order of the β*-globin* genes and the LCR could only be explained by looping (Grosveld et al., 1987; Hanscombe et al., 1991; Dillon et al., 1997). A few years later, the effect of natural mutations by defective enhancers located at very long distance, like in the case of polydactyly, was very difficult if not impossible to explain by mechanisms other than looping (Lettice et al., 2003).

The regulation of the β*-globin* like genes by its LCR, was and still is the best-studied example for the looping model (Figure 3C; Grosveld et al., 1987). In adults, the LCR and the β*-globin* promoter are located in close proximity contributing to the formation of new chromatin loops by the recruitment of specific TFs such as LDB1, TAL1, GATA1 and KLF1 to the LCR (Noordermeer and de Laat, 2008; Palstra et al., 2008a). The different enhancer elements and the gene appear to form a regulatory hub where all the different elements appear to interact with each other (Allahyar et al., 2018). Interestingly, even though the individual enhancers appear to interact, the overall activity of the LCR usually appears to be the result of an additive effect of the individual enhancer elements rather than a synergizing effect, with the individual enhancers exhibiting different properties (Fraser et al., 1993; Bender et al., 2012). Absence of crucial TFs in the LCR results in the disruption of chromatin conformation and in gene mis-expression.

Recent allele specific interaction studies indicate that the LCR interacts with more than one of the (mouse) β*-globin* genes simultaneously (Allahyar et al., 2018), whereas previous studies showed that only the (human) β*-globin* gene can be active at any given moment in time in the situation where two genes are in contact with the LCR at the same time (the γ*-* and β*-globin* genes in human and the β*maj-* and β*minor-*globin in mouse) (Wijgerde et al., 1995; Trimborn et al., 1999). These observations lead to the conclusion that transcription is a discontinuous process and that the frequency and stability of the promoter-enhancer interactions is a very important parameter in determining the level of transcription. The observation that the mouse LCR would interact with two β*-globin* genes simultaneously, but that only one would be expressed, sets up the interesting question whether this is perhaps particularly prevalent among genes “competing” for the same enhancers.

Looping interactions are not limited only to enhancers and promoters. Subsequent studies suggest that enhancers make contacts also with gene bodies following the elongating RNAPII (Lee et al., 2015). In parallel, Polycomb proteins (PRC1, PRC2) facilitate the regulatory topology by repressing genes through chromatin interactions and keep them under tight control (Schoenfelder et al., 2015b; Cruz-Molina et al., 2017; Cai et al., 2021). Moreover, some promoters (E-promoters) can act as bona-fide enhancers and are in close proximity with others to activate gene expression (Dao et al., 2017).

An interesting debate is whether gene activation precedes locus conformation or *vice versa* (van Steensel and Furlong, 2019). In a previous study, during neuronal differentiation, promoter–enhancer interactions appeared along with changes in gene expression (Bonev et al., 2017). However, during erythropoiesis, chromatin structure precedes expression and does not require the presence of TFs, but TFs are essential for the advancement to, or maintenance of, a fully functioning active chromatin hub (Drissen et al., 2004). Moreover, chromatin loops are not altered in the β*-globin* locus upon transcriptional inhibition, suggesting that structure precedes function (Palstra et al., 2008b). Interestingly, the recruitment of LDB1 to the β*-globin* promoter depends on GATA1, in contrast to its recruitment to LCR. In GATA1-null cells that do not express β*-globin*, its expression can be rescued by the tethering of LDB1 via a zinc finger domain to its promoter, mediating its interaction with the LCR, and thus supporting the hypothesis that conformation comes first (Deng et al., 2012). In another study LDB1 was directed to the silenced promoter of the embryonic β*-like globin* (β*h1*) gene in adult mice erythroblasts (Deng et al., 2014). In parallel, during the zygotic genome activation, the formation of TADs coincides with the onset of gene expression (Hug et al., 2017).

### Pre-looping (Pre-determined Contacts)

Recent studies propose an additional way on how chromatin conformation controls gene transcription. Genes are often in close proximity to their cognate enhancers without being actively transcribed. Although their cognate enhancer is often bound by various TFs, it lacks the binding of a crucial TF required for gene activation (Kolovos et al., 2016). At the same time, RNAPII is stalled at the promoter (Ghavi-Helm et al., 2014). In that case, when a developmental or a differentiation signal triggers the additional recruitment of crucial TF(s) to the enhancer, looping is maintained and transcription is induced. This model is termed pre-looping (Figure 3D; Ghavi-Helm et al., 2014; Kolovos et al., 2016; Rubin et al., 2017). During mouse development, pre-existing chromatin contacts of the Hox genes could help in the recruitment of the necessary transcription factors, in order tissue-specific promoter-enhancer interactions to occur (Lonfat et al., 2014). Moreover, loops mediated by the PRC1 and PRC2 complexes in pluripotent cells are not only repressing the genes inside such loops, but also maintain them in close proximity with their regulatory elements permitting a fast response (activation) to specific differentiation signals (Figure 3E; Schoenfelder et al., 2015b; Cruz-Molina et al., 2017). Similarly, the CTCF and cohesin complex bring the *Shh* promoter and ZRS enhancer in close proximity in posterior and anterior limbs (Paliou et al., 2019). Although they are in close proximity, the *Shh* gene is differentially expressed in these tissues (Williamson et al., 2016). An even closer proximity is observed when *Shh* is activated in the posterior limbs (Williamson et al., 2016). As it is clear from the previous examples, specific topological features are not a sufficient criterium to initiate transcription (Ghavi-Helm et al., 2014; Hug et al., 2017).

Most of the interactions of the pre-looping model are not mediated or predicted by CTCF, but by TFs and RNAPII, e.g., in HUVEC cells, *SAMD4A* is not expressed while its promoter is in close proximity with its enhancers. Upon activation by TNFα signaling, the TF NFκB is released from the cytoplasm, enters the nucleus and binds to the enhancer leading to looping maintenance and the activation of *SAMD4A* expression (Kolovos et al., 2016). Other examples of pre-looping were later reported in macrophages, upon adipogenesis, differentiation of the epidermis, during differentiation of mouse embryonic stem cells (ESCs) to neural progenitors, in the mouse *HoxB* locus and in hypoxia, but also as a mechanism of action for specific transcription factors like PAX5 (Barbieri et al., 2017; Cruz-Molina et al., 2017; Rubin et al., 2017; Siersbæk et al., 2017).

Thus, there is an interesting conundrum. How could transcription be controlled by two different chromatin conformations; looping and pre-looping. According to the pre-looping model, loops formed by CTCF, cohesin, PRC1 or PRC2 could contain poised enhancers and promoters in close proximity only to activate them with subsequent tighter contacts, e.g., after post translational modifications of essential for activation TFs take place (Figures 3D,E; Drissen et al., 2004; Robson et al., 2019). According to the looping model, loops appear and disappear dynamically during development, in parallel with transcriptional activation and could flexibly fine-tune transcription (Javierre et al., 2016; Bonev et al., 2017). Another explanation could be that most of the looping paradigms are studied in steady-state systems or when comparing only two stages of differentiation or development (Grosveld et al., 1987; Palstra et al., 2008a; Deng et al., 2012; Kolovos et al., 2014). Maybe some genes have been selected evolutionary to use one of the two ways of chromatin conformation. However, studying more than two stages of differentiation, development or embryogenesis could unveil which of the two mechanisms is used mostly (Stadhouders et al., 2018; Di Stefano et al., 2020). Although the dynamics of nuclear organization have been studied so far during mitosis (Naumova et al., 2013), meiosis (Patel et al., 2019), hormone treatment, differentiation (Bonev et al., 2017) and cell reprogramming (Stadhouders et al., 2018), there is an immediate need for methods that are precisely tailored for the study of time-dependent conformational changes (4D) (Di Stefano et al., 2020).

## Transcription Factories as the Driving Force of Transcription

The established transcription model claims that the polymerase moves along the DNA sequence to produce the transcript. Nowadays, it is believed that transcription takes place in nucleoplasmic hot spots (called “transcription factories” Papantonis and Cook, 2013, see above), mediated by a high local concentration of all the necessary transcription factors. This notion suggests that the polymerase is located primarily, but not fixed in “transcription factories” (Ghamari et al., 2013; Papantonis and Cook, 2013). In the traditional model of transcription, RNAPII leaves the promoter and moves along the DNA template. In “transcription factories”, the RNAPII is present these nucleoplasmic hotspots, while genes and their respective promoters diffuse to them, as transcription takes place through the movement of the DNA template via transcription factories (Jackson et al., 1981; Iborra et al., 1996; Papantonis et al., 2010; Cho et al., 2018). Notably a similar type of mechanism/principle has been proposed for “loop extrusion”, the mechanism by which loops are formed and where the DNA moves through the cohesion complex (see above). Time course experiments indicated that the enhancer and the promoter of the *Cd47* and *Kit* genes are in close proximity during transcription (Lee et al., 2015). “Transcription factories” are most likely a collection of several “active chromatin hubs,” that merge in a phase transition type process containing several polymerase complexes, each transcribing a different template (de Laat and Grosveld, 2003; Larson et al., 2017).

Current interests are focused on liquid-liquid phase separation (LLPS) as the driving force to concentrate the necessary elements (e.g., enhancers, transcription factors, RNAPII, etc.) at active chromatin hubs or transcription factories (Sabari et al., 2018; Guo et al., 2019; Nair et al., 2019). The concept of phase transition, LLPS is a mechanism to generate “structures” without membranes (Hildebrand and Dekker, 2020). Molecular seeds are thought to start the process of phase transition leading to a local enrichment of protein-protein complexes. Intrinsically disordered protein domains are thought to play a major role by their ability to have multivalent interactions (multi-modular features) (Li P. et al., 2012). It has been shown that artificial condensates are able to physically pull together specific loci, and thus, LLPS generate mechanical force to the chromatin (Shin et al., 2018). Such compartmentalized hydrogel-like states would have a reduced fluidity and movement of proteins, which would for example fit with the concept that the DNA moves through the polymerase in a transcription factory rather than the polymerase moving along the DNA. Subsequent research has revealed that the Mediator complex, along with other transcription factors, coactivators, and RNAPII, form condensates during transcription (Boija et al., 2018; Cho et al., 2018; Chong et al., 2018; Sabari et al., 2018; Guo et al., 2019). Phase-separated HP1α and RNAPII showed the ability to create phase-separated heterochromatin and euchromatin droplets, respectively (Larson et al., 2017; Lu et al., 2018). Condensation of bound TFs and coactivators is induced by multivalent enhancer sequences via LLPS (Shrinivas et al., 2019). Although this idea has not been thoroughly tested, it has been observed that LLPS causes enhancers that would typically dwell in distant TADs to migrate closer (Nair et al., 2019). The local concentration of RNA can impact condensate formation and dispersion, acting as a transcriptional feedback mechanism (Henninger et al., 2021).

It has also been proposed that the outer edge of phase-separation droplets acts as a barrier that proteins could not pass through (Strom et al., 2017), despite the quick recovery of CDK9-mCherry signal after photo-bleaching, suggesting that CDK9-mCherry is constantly recruited to the stably positioned transcription factories (Ghamari et al., 2013), Chromatin compartmentalization might be the reason that activating transcription factors are not present in B compartments (Laghmach and Potoyan, 2021). Phase separation could explain several confusing observations, like how transcriptional activation occurs without direct physical contact between enhancers and promoters through eRNAs (Cai et al., 2020), or multi-enhancer and multi-promoter contacts (Li G. et al., 2012; Jin et al., 2013), or simultaneous regulation of more than one gene by a single enhancer (Fukaya et al., 2016). In parallel, recent data suggest that forces other than the ones derived from LLPS could also stabilize transcription factories (Ulianov et al., 2021).

The β*-globin* active chromatin hub, containing *Hbb-b1*, its LCR (60 Kbp upstream of *Hbb-b1*) and *Eraf* (encoding an α-globin stabilizing protein, located ∼25 Mbp far from *Hbb-b1*) is the best example of different genes in the same transcription factory. Various assays, like 3C-like methods, RNA and DNA FISH coupled to immuno-labeling, confirmed that *Hbb-b1*, its LCR and *Eraf* are found together in sites rich with RNAPII (Bender et al., 2012; Mitchell et al., 2012). As mentioned above, another property of transcription factories is that they encompass groups of genes (located *in cis* or *in trans*), which are co-regulated by specific signaling pathways or activators leading to the idea that co-regulated genes are expressed in “specialized” transcription factories (Schoenfelder et al., 2010). This is corroborated by ChIA-PET of active RNAPII, which uncovered spatial associations between co-regulated and co-transcribed genes in response to various stimulations (Papantonis et al., 2012; Li et al., 2015). Moreover, RNAPII transcribed genes are located in separate factories than RNAPIII genes. TNFα responsive genes and erythropoietic genes are also located in distinct factories (Pombo et al., 1999; Papantonis et al., 2010; Schoenfelder et al., 2010; Baù et al., 2011; Monahan et al., 2019). Therefore, it is tempting to conclude that there are “specialized” transcription factories.

## The Interplay Between Structure and Function Through Development, Differentiation and Evolution

Loops within the genome can be separated into two categories according to their role (Kolovos et al., 2014); structural and functional. Structural loops are forming the building blocks of the 3D conformation of the genome. They can take place between DNA segments (none of which is a promoter or an enhancer) through CTCF or cohesin binding, forming large TAD domains with their base defining the domain boundaries (Dixon et al., 2012; Rao et al., 2014; Zuin et al., 2014). Various chromatin conformation capture results suggest that these structural loops are the same between different cell types (Dixon et al., 2015; Schmitt et al., 2016). Therefore, structural loops could contribute indirectly to the regulation of gene expression, via the formation of TADs confining genes and their respective regulatory elements in a dedicated 3D nuclear space. Functional loops, which often appear within structural loops, are the ones bestowing a function/task (activation/repression/poised) to a gene and often correspond to sub-TADs (Grosveld et al., 1987; Splinter et al., 2006; Palstra et al., 2008a; Wendt et al., 2008; Kagey et al., 2010; Schoenfelder et al., 2010; Kolovos et al., 2012; Phillips-Cremins et al., 2013; Sofueva et al., 2013; Fang et al., 2014; Ghavi-Helm et al., 2014; Rao et al., 2014; Zuin et al., 2014; Ji et al., 2016; Kolovos et al., 2016; Phanstiel et al., 2017; Rubin et al., 2017). These interactions could be direct or indirect. The direct interaction is between two DNA segments with one containing a regulatory element (an enhancer or a silencer) and the other the promoter of the target gene (de Laat and Grosveld, 2003; Palstra et al., 2008a; Stadhouders et al., 2012; Kolovos et al., 2014; Kolovos et al., 2016). The indirect interaction is between an enhancer/silencer and a DNA segment which is not the promoter of the target gene, which subsequently interacts with the promoter creating an active regulatory hub (Stadhouders et al., 2012; Schuijers et al., 2018; Quinodoz et al., 2018). An example is the *Myc* loci where its super-enhancer interacts with its promoter through a CTCF site located 2 Kbp upstream of the *Myc* promoter (Schuijers et al., 2018), similar to the way *Myb* is regulated in mouse erythroid cells (Stadhouders et al., 2012).

**FIGURE 4:**
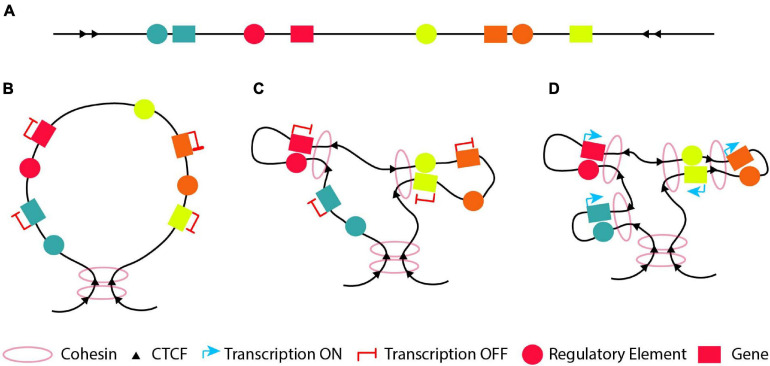
The “loop within loops” model. **(A)** An example of a DNA segment which contains four genes (blue, red, green and orange) depicted with boxes and their cognate regulatory elements (circles with the respective colors). **(B)** A structural loop of 1-2Mb forms a TAD with its base to define the domain boundary. **(C)** At an early developmental/differentiation stage most genes are silenced. Thus, inside the structural loop, the genes will either not form any loops with their cognate regulatory element (looping model; blue and orange genes) or form functional silencing-loops within structural-loops (loops-within-loops) with their cognate regulatory element lacking a crucial TF (pre-looping model; red and green genes). **(D)** At later developmental stages, new functional loops are formed within the pre-existing functional loops (orange gene) and/or the structural loop (blue gene) forming “loops within loops” in order to activate the orange and blue genes, respectively. At the same time, the previously pre-looped genes (red and green) are activated as a result of a recruitment of the necessary TF to their cognate enhancer or due to conversion of their cognate poised enhancer to an active one.

In this part, we describe how functional and structural loops are formed, as well as the shape of the 3D chromatin organization at different stages of development and differentiation (Figure 4). As already mentioned before, loops are critical for proper gene expression and the integrity of these loops is indispensable for the development of various tissues, differentiation of cells, diseases and cancer. Hence, it is important to understand how or even when they are formed in order to decipher how the local chromatin architecture contributes to different phenotypes.

The chromatin architecture changes significantly during gametogenesis and early embryonic development (Li et al., 2019; Zheng and Xie, 2019). In short, during spermatogenesis A/B compartments and TADs vanish in pachytene spermatocyte and then reappear in round spermatid and mature sperm stages (Wang et al., 2019). The transcriptionally inactive mouse sperm displays chromatin conformation features, with CTCF and cohesin occupying positions similar to those in mESCs, implying the important role of these factors in shaping chromatin conformation even in the absence of transcription (Carone et al., 2014; Du et al., 2017; Jung et al., 2017). Those features, albeit weaker, were also detectable in oocytes (Gassler et al., 2017). During oogenesis, the oocyte shows the typical higher-order structures until the germinal vesicle (GV) stage (Flyamer et al., 2017). The strength of those features declines dramatically from the immature oocytes to mature oocytes (Flyamer et al., 2017), and from this point forward oocytes lack the typical interphase chromatin structures (Du et al., 2017; Ke et al., 2017). Chromatin structure at this point resembles the chromatin structure during mitosis (Naumova et al., 2013).

After fertilization, chromatin conformation undergoes dramatic reprogramming (Zheng and Xie, 2019). Since TADs and A/B compartments are very weak in early-stage mouse embryos, some studies have shown that chromatin adopts a more relaxed state (Du et al., 2017; Ke et al., 2017). However, loops and TADs have also been observed in mouse zygotes (Gassler et al., 2017). Indeed, TADs are maintained during the oocyte-to-zygote transition in mice and gradually become more prominent (Du et al., 2017; Ke et al., 2017). Genes are initially silenced, but after the zygotic genome activation (ZGA), they are activated (Clift and Schuh, 2013). ZGA occurs in the 2-cell embryo in the mouse (Du et al., 2017; Ke et al., 2017). Inhibition of ZGA did not prevent the formation of TADs (Ke et al., 2017), suggesting that TAD formation precedes their main function of transcriptional control (Flyamer et al., 2017; Hug et al., 2017; Ing-Simmons et al., 2021). Thus, TADs act first as building blocks of architecture and then as transcriptional controllers. TADs are established in Drosophila during ZGA. Compartmentalization of the chromosomes at the zygote stage seems to be driven by a different mechanism than the one of TAD formation (Flyamer et al., 2017; Ke et al., 2017). Specifically, the paternal originated chromosomes maintain all the genome structures, whereas the maternal chromosomes lose the A/B compartments. During the two-to-eight-cell stage, conformation is slowly re-established and become progressively stronger in both, maternal and paternal chromosomes (Du et al., 2017; Flyamer et al., 2017; Gassler et al., 2017; Ke et al., 2017).

Common TADs and A/B compartments that correspond to transcriptionally active regions are present in both pluripotent cells and differentiated cells, but the chromatin of pluripotent cells is less compacted than in other cell types (Melcer and Meshorer, 2010; Gaspar-Maia et al., 2011). In pluripotent cells, pluripotency TFs are found in the same areas of the nucleus, establishing long-range chromatin interactions with each other (Bouwman and de Laat, 2015). The observation that gene loci controlled by pluripotency factors are located in close proximity inside the nucleus, suggests a regulatory mechanism similar to phase separation (De Wit et al., 2013). For example, it was shown that many KLF4-bound regions are in close proximity to each other in pluripotent cells and released upon differentiation or KLF4 depletion (Wei et al., 2013).

Early in differentiation, pluripotent genes are initially repressed and subsequently activated (Phillips-Cremins et al., 2013). Early differentiation genes exhibit a permissive architecture and are in close proximity to their associated poised enhancers. Upon differentiation, their enhancers become active and activate their target gene(s) (Cruz-Molina et al., 2017). This suggests that conformation structures mediated by Polycomb proteins create a permissive regulatory environment, where poised regulatory elements are ready to be expressed (Cruz-Molina et al., 2017). Similar observations have been also made in other differentiation pathways, such as adipogenesis (Siersbæk et al., 2017).

An intriguing question is how regulatory elements are generated during evolution, because it is clear that a gene can use different regulatory elements in different cell types or during differentiation to more mature cell types. Interestingly, neocortical enhancers start out as basic proto-enhancers and evolve in complexity and size over time (Emera et al., 2016). Moreover, the rapid evolution of enhancers in liver across 20 mammalian species (*18 placental species from Primates, Rodents, Ungulates, Carnivores and 2 marsupial species*) is a general feature of mammalian genome as observed by profiling genomic enrichment of H3K27ac and H3K4me3 of liver enhancer regions (Villar et al., 2015). Interestingly, the majority of the recently evolved enhancers are derived from ancestral DNA exaptation and are significantly over-represented in the vicinity of positively selected genes in a species-specific manner (Villar et al., 2015). Thus, it would be tempting to speculate that species, which were less “evolved”, have developed “simpler” regulatory elements to control their gene expression. Since these species were more primitive, gene expression profiles were less complicated and more specific for each of the much smaller number of different cell types. During evolution and the appearance of more complex organisms that require an increased diversity of cell composition, the control of gene expression became more complex and new regulatory elements appeared (Ong and Corces, 2011).

Thus, during the early stage(s) of development, differentiation or evolution, a DNA segment with various genes and regulatory elements (Figure 4A) will mostly form structural loops to shape the chromatin (Figure 4B), since chromatin conformation during ZGA is independent of activation of gene expression (Hug et al., 2017; Ing-Simmons et al., 2021). At an early developmental/differentiation stage or during the oocyte-to-zygote transition, genes are often silenced. Based on the pre-looping model, some genes will already be in close proximity with their enhancer, which lacks one or more necessary TFs and is in a poised state (Figure 4C, red and yellow genes and their respective regulatory elements) to promote their activation or silencing, forming functional “loops-within-loops”. In parallel based on the looping model, the genes will be far apart from their cognate enhancer in the 3D space (Figure 4C, green and orange genes and their respective regulatory elements). At a later developmental/differentiation stages, genes which do not have a poised functional loop, will have to form new functional loops within the pre-existing structural or silencing-functional loops (“loops-within-loops”) in order to become transcriptionally active (Figure 4D green and orange genes and their respective regulatory elements). At the same time, the previously silenced genes in a poised loop (Figure 4D, red and yellow genes) are activated as a result of a recruitment of the necessary TF to their cognate enhancer or due to conversion of their cognate poised enhancer to an active one.

**FIGURE 5:**
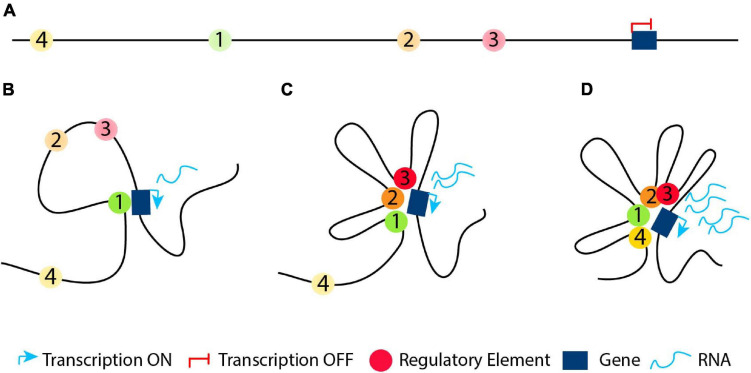
Regulatory elements in development, differentiation or evolution. **(A)** At an initial stage in development, differentiation or evolution, the genome has a silenced gene and a regulatory element located in a distance from it (regulatory element 1, depicted with a green circle). **(B)** The original “early” regulatory element interacts with its target gene in order to activate it. **(C)** At subsequent developmental, differentiation or evolutional stages, the genome could develop new regulatory elements (regulatory elements 2 and 3, depicted with orange and red circle, respectively) between the gene and its original “early” regulatory element, which interact with their target gene. **(D)** At some cases, at later developmental, differentiation or evolutional stages, we could observe a new regulatory element (regulatory element 4, depicted with a yellow circle) outside the original “early” loop, which could also interact with its target gene via a formation of a new loop.

In this context, at initial stages of development, differentiation or evolution, we speculate that the genome must have an initially regulatory element located at a distance from its target gene (Figure 5A, green regulatory element), which interacts with its target gene via a specific loop (Figure 5B). At a subsequent developmental, differentiation or evolutional stage, we hypothesize that new (cell/tissue type specific) regulatory elements are developed between the gene and its original “early” regulatory element, which can interact with their target gene (Figure 5C, orange and red regulatory elements). Thus, we could observe an initial big loop, which can be functional or either structural, containing other loops at later stages. The latter will form new “loops-within-loops” to accommodate new expression patterns. This type of regulation is observed when comparing the activity of different regulatory elements in multiple stages of differentiation/development/evolution (de Laat and Grosveld, 2003; Palstra et al., 2008a; Mylona et al., 2013; Pimkin et al., 2014; Villar et al., 2015; Goode et al., 2016). However, we cannot exclude the possibility that in rare cases during development, differentiation or evolution, a regulatory element outside the original “early” loop would develop, which could also interact with its target gene at subsequent stage (Figure 5D, yellow regulatory element). Finally, all these aforementioned interactions could satisfy either the pre-looping or the looping model (Figure 3). In an evolutionary sense, developing novel enhancers is an almost inevitable feature of multicellular organisms with different cell types and functions. Other mechanisms are very difficult to envision for the enormous diversity in gene expression patterns, which is ultimately due to the fact that DNA is a linear molecule.

## Chromatin Conformation From Early Development to Differentiation

The internal structure of TADs becomes more organized during development and differentiation, as TADs enable more enhancer-promoter contacts (Bonev and Cavalli, 2016). This is important during development, where specific genes need to be activated or repressed to promote specific cell programs and lineage commitment. For example, during limb development, the *HoxD* cluster is located at the border of two flanking regulatory elements, which are contained into two separated TADs (Lonfat and Duboule, 2015). In the beginning, the 3′ TAD is active and regulates the proximal patterning. Subsequently, this TAD is switched off while the 5′ TAD becomes active and controls distal structure. Activation of *Hox13* switches off the 3′ TAD through a global repressive mechanism and interacts with enhancers at the 5′ TAD that sustains its activity (Beccari et al., 2016). Thus, the *HoxD* cluster contains a dynamic TAD boundary, regulating the switching between the flanking TADs and enabling a proper limb development (Rodríguez-Carballo et al., 2017).

Early studies before the discovery of TADs, showed that the lack of CTCF or its disruption on one of the binding sites in the mouse β*-globin* locus resulted in an altered interactome (Splinter et al., 2006). New insights in the significance of TADs during development came from a study of the *HOXA* locus, which is important for development of many tissues such as limb. The *HOXA* locus is organized in two different TADs, with CTCF and cohesin binding sites at their boundary. The disruption of CTCF or Cohesin recruitment at the boundary sites of these two TADs allows the spreading of euchromatin into heterochromatin domains and the subsequent ectopic activation of *HOX* genes during cell differentiation due to new chromatin contacts (Zuin et al., 2014; Lonfat and Duboule, 2015). Another example is the *Tfap2c* and *Bmp7* locus, which is split into two functional and structural domains, with each gene being present in separate TADs with their cognate enhancers. Inversions at the TAD boundary, changes the position of *Bmp7*’s cognate enhancer into the TAD containing *Tfap2c*, thus leading to upregulation of the latter gene and downregulation of Bmp7 (Tsujimura et al., 2015). This illustrates the extent to which proper topology influences the regulation of expression of developmentally essential genes. A fine example of regulatory specificity of enhancers controlled by chromatin architecture is that of *Pitx1*, a regulator of hindlimb development (Kragesteen et al., 2018). In hindlimbs, *Pitx1* is in close proximity with its enhancer (active), allowing for normal leg morphogenesis. In forelimbs, *Pitx1* is physically separated from the enhancer (inactive), allowing for normal arm development. The disturbance of that specificity (e.g., due to structural variants) can cause gene mis-expression and disease *in vivo* (Kragesteen et al., 2018). Transcription after activation of the glucocorticoid receptor occurs without significant changes of the pre-looped chromatin interactions, enabling its rapid reaction (Hakim et al., 2011). Changes in chromatin topology and conformation have already been associated and described in muscle progenitor specification and myogenic differentiation (Zhang et al., 2020), sensory experience during post-natal brain development (Tan et al., 2021), dendritic cell development and differentiation (Chauvistré and Seré, 2020), and neural development (Kishi and Gotoh, 2018).

An interesting question is whether conformation accompanies cell lineage decision and what the role of TFs is. During reprogramming, TFs reorganize genome structural features before changes in gene expression occur (Stadhouders et al., 2018). Somatic cell reprogramming is a useful model for investigating how genome topology affects cell fate decisions. A recent study, investigating chromatin interactions in ESC, iPSC and NPCs, revealed that reprogramming does not completely restore a number of pluripotency-related interactions (Beagan et al., 2016). CTCF was abundant in these regions in ESC, while poor in differentiated NPCs. CTCF binding was not restored in iPSC causing an inadequate pluripotent genome topology recovery. The embryonic and trophoblast lineages have significant differences between them, in their epigenetic landscapes and their 3D conformation (Schoenfelder et al., 2018). ESCs have an enrichment for repressive interactions between gene promoters and also involving poised/silenced enhancers (marked with H3K27me3), whereas trophoblasts have an enrichment for active enhancer-gene interactions (Schoenfelder et al., 2018). Similarly, during neuronal differentiation of ESCs, Polycomb repressive complex 1 and 2 (PRC1 and PRC2) are known to have important functions in mediating repressing interactions. PRC1 mediated interactions are disrupted and gene-enhancer interactions become prominent (Bonev et al., 2017). Interestingly, poised enhancers in ESCs are already in close proximity with their target genes in a PRC2 dependent manner (Ngan et al., 2020). Deletion of PRC2 core components leads to activation of their target genes and embryonic lethality (Boyer et al., 2006; Bracken et al., 2006). When these enhancers are activated during differentiation of ESCs to neural progenitors, the interaction with their cognate genes is preserved, leading to their activation (Cruz-Molina et al., 2017). This is similar to the aforementioned pre-looping phenomenon. All in all, these results demonstrate that chromatin architecture changes may not cause instant transcriptional changes. As an alternative, structure seems to set the stage for future transcriptional changes by sculpturing the chromatin environment.

X chromosome inactivation (XCI) is another well-studied example to show how the 3D chromatin organization impacts development, as well as the differences between the two homologs (Lee and Bartolomei, 2013). One of the two X-chromosomes in female cells is randomly inactivated to equalize the expression levels of the X-linked genes between female and male cells, early during embryonic development or upon differentiation of female ESCs (Gribnau and Grootegoed, 2012). Several regulatory elements and genes directing the XCI process are located in a small region, the X inactivation center (Xic) (Barakat et al., 2014). This region harbors the best-studied mammalian lncRNA, *Xist* and its negative regulator *Tsix* (Lee et al., 1999). While *Xist* silences one X chromosome *in cis*, *Tsix* represses *Xist* also *in cis* and thus these two lncRNAs form a regulatory switch locus (Nora et al., 2012). The *Xist* locus has been proposed to be organized in two big TADs and XCI is initiated by the upregulation of *Xist* in one of the two X-chromosomes (Chaumeil et al., 2006; Engreitz et al., 2013). In another study, the two X chromosomes were shown to have distinct and different chromatin organization. The active X presented distinct compartmentalization of active and inactive regions, while the inactive X compartments were more uniform (Tan et al., 2018). TADs were present in the active X chromosome, but not in the inactivated X chromosome (Splinter et al., 2011; Nora et al., 2012; Giorgetti et al., 2016). In comparison, two mega-structures appeared on the inactivated X chromosome, separated by a microsatellite repeat containing several CTCF-binding sites (Horakova et al., 2012; Rao et al., 2014; Wang et al., 2016). Interestingly, a study using mathematical prediction and experimental validation suggested that three internal elements (CTCF/binding sites within the *Linx*, *Chic1* and *Xite/Tsix* loci) might work in partnership with boundary elements for the formation and the stabilization of the two TADs (Bartman and Blobel, 2015). The deletion of these internal elements is sufficient to disrupt the TADs and subsequently triggers ectopic expression of genes at the neighboring TAD hence disturbing XCI process (Nora et al., 2012; Dixon et al., 2016).

Overall, TAD formation and maintenance as well as specificity of the enhancer-promoter interaction play key roles during development and differentiation to ensure the finely tuned regulation of gene expression and lineage decision.

## Chromatin Conformation in Disease and Cancer

Human diseases are often caused by structural variations (SVs) in the genome, through disruption of genes or changes in gene dosage (Spielmann et al., 2018; Ibrahim and Mundlos, 2020). While their effect in coding regions can be easily predicted, their occurrence in non-coding regions requires further investigation to address its influence on gene expression, for example in the case of limb formation involving the TAD-spanning *WNT6/IHH/EPHA4/PAX3* locus (Lupiáñez et al., 2016). SVs have the potential to interfere with genome architecture causing disease phenotypes (Lupiáñez et al., 2015; Spielmann et al., 2018). Depending on the type but also the extent of the SV, the effect on gene regulation may vary a lot (Ibrahim and Mundlos, 2020).

Disruption of genome architecture may lead to altered gene expression in a variety of ways and, as a result, disease phenotypes. This disruption is separated into inter-TAD and intra-TAD alterations.

Inter-TAD alterations can disrupt and rewire the 3D chromatin architecture resulting in changes of TAD boundaries, mis-regulation of important genes with deleterious effects and relocation of regulatory elements such as enhancers and/or silencers. Inter-TAD alterations are caused by many reasons. Genome architecture disruption involves the disruption of TADs borders, leading to contacts of enhancers and genes, otherwise insulated from each other, and thereby, the ectopic activation of those genes. This phenomenon is called ‘’enhancer adoption” or ‘’enhancer hijacking” (Table 1; Lettice et al., 2011; Northcott et al., 2014; Lupiáñez et al., 2016; Kaiser and Semple, 2017). Deletions result in TAD fusion (Table 1; Katainen et al., 2015; Lupiáñez et al., 2015; Flavahan et al., 2016), inversions in a swap of DNA regions (TAD shuffling) and duplications or translocations of regulatory or structural elements in new domains (neo-TADs) (Table 2; Gröschel et al., 2014; Northcott et al., 2014; Lupiáñez et al., 2015; Franke et al., 2016; Weischenfeldt et al., 2017). Furthermore, inter-TAD alterations could be caused by inversions, translocations of regulatory elements which may result in gain-of-function events by coupling enhancers with newly associated promoters, or loss-of-function events by separating enhancers from their associated promoters or a combination of the two (Table 2; Lupiáñez et al., 2016; Spielmann et al., 2018). To mention here, that while the above studies stress out the insulating role of TAD boundaries, it is important to keep in mind that TAD boundaries may not be the only component needed to maintain them (Anania and Lupiáñez, 2020). TADs did not fuse completely after serial deletions at the *Sox9* locus. This occurred only after the deletion of other CTCF sites within the locus (Despang et al., 2019). Deletions of CTCF-binding sites at the *Shh* locus result in structural changes, but TAD insulation is maintained (Paliou et al., 2019). Overall, these results reveal the ability of TAD borders to successfully organize the genome into distinct regulatory domains, as well as their ability to work and communicate with the internal structure elements.

**TABLE 1:** Summary of inter-TAD alterations (Enhancer adoption and TAD fusion), the disease/abnormality they caused and their description.

*Enhancer adoption*		
*LMNB1 locus*	Adult-onset demyelinating leukodystrophy (ADLD)	A deletion eliminates a TAD boundary, leading to new interactions between the *LMNB1* promoter and three non-cognate enhancers and its subsequent activation, resulting in the progressive central nervous system demyelination (Giorgio et al., 2014)
*FOXG1 locus*	Rett syndrome	A telomeric deletion, including the TAD border, results in the ectopic activation of *FOXG1* by active enhancers in the brain (Allou et al., 2012)
*GFI1B locus*	Medulloblastoma	Somatic structural variants place *GFI1* or *GFI1B* near active enhancer sites, resulting in activation (Northcott et al., 2014)
*SNCAIP locus*	Group 4 medulloblastomas	A duplication of *SNCAIP* gene results in the ectopic activation of the putative oncogene *PRDM6* (Arabzade et al., 2020)
***TAD fusion***		
*EPHA4 locus*	Brachydactyly	Deletions in the *EPHA4* locus that include a TAD border result in a fusion of the neighboring TADs, which attaches a cluster of limb-associated *EPHA4* enhancers to the *PAX3* gene and its concomitant mis-expression (Lupiáñez et al., 2015)
*Six TAD boundaries encompassing T-ALL related genes*	T-cell acute-lymphoblastic leukemia (T-ALL) or medulloblastoma	TAD disruption leads to ectopic proto-oncogene activation and abnormal cell proliferation (Northcott et al., 2014; Hnisz et al., 2016b; Weischenfeldt et al., 2017). CRISPR-engineered deletions of the TAD boundaries near the known oncogenes *TAL1* and *LMO2* result in new interactomes of those oncogenes with distal enhancers, leading to their aberrant activation (Hnisz et al., 2016b)
*NOTCH1 locus*	Ovarian cancer	Downregulation of *NOTCH1* gene due to its altered interactome as a result of mutations in the CTCF sites that disrupt the TAD boundary (Ji et al., 2016)
*Various CTCF binding sites*	Colorectal cancer	Frequently mutated *CTCF binding sites* lead to TAD boundary disruption and altered interactomes between genes and their regulatory elements (Katainen et al., 2015)
*IRS4 locus*	Lung squamous carcinoma, sarcomas and cervical squamous carcinoma	Deletions occurring at TAD boundaries coinciding with CTCF recruitment downstream of the *IRS4* locus led to *IRS4* overexpression (Weischenfeldt et al., 2017)
*NEK6 locus*	B cell lymphoma cell lines	Deletion of all CTCF-binding sites in the *NEK6* super-enhancer borders decreased the expression of *NEK6* while increased the expression of the neighboring *LHX2* gene (Huang et al., 2017)

**TABLE 2:** Summary of inter-TAD alterations (TAD shuffling, Inter-TAD loss- or gain-of-function alterations and Neo-TADs), the disease/abnormality they caused and their description.

*TAD shuffling*		
*Wnt6/Epha4 locus*	F-syndrome	An inversion at the *Wnt6/Epha4* locus that misplaces the *Epha4* enhancers near *Wnt6* gene, causing its mis-expression in the developing limb bud (Lupiáñez et al., 2015; Kraft et al., 2019)
*Ihh/Epha4 locus*	Polydactyly	Duplications of the previous enhancers and rearranging them in front of the *Ihh* gene induce overexpression of *Ihh* (Kraft et al., 2019)
*TFAP2A locus*	Branchio-oculofacial syndrome	Inversion of the *TFAP2A* TAD resulted in lower *TFAP2A* expression due to the fact that the promoter was separated from its associated enhancers (Laugsch et al., 2019)
*Shh locus*	Digit syndactyly	An inversion at the *Shh* locus places the *Shh* gene in a TAD together with a limb enhancer, that induces its activation (Lettice et al., 2011)
*MEF2C locus*	5q14.3 microdeletion syndrome	Patients with balanced *MEF2C* translocations have been shown to be affected by the separation of promoters from their associated enhancers. The influence of these translocations was confirmed in patient-derived LCLs, which showed lower *MEF2C* expression (Redin et al., 2017)
*GATA2 locus*	Acute myeloid leukemia sub-types	A chromosomal inversion and translocation in chromosome 3 at two different breakpoints place the *GATA2* enhancer in the same TAD as the *EVI1* oncogene. The enhancer is then in close proximity with the *EVI1* promoter triggering its activation, which is responsible for the development of the disease (Gröschel et al., 2014)
*IGF2 locus*	Colorectal cancer	Recurrent tandem duplications encompassing a TAD boundary result into new interactions between *IGF2* and a cell specific super-enhancer located in the adjacent TAD, leading to its > 250-fold overexpression (Weischenfeldt et al., 2017). The duplications in the abovementioned TAD boundary are tandem rather inverted or dispersed, suggesting that the orientation of the enhancer and *IGF2* is probably important for the activation of *IGF2* (Beroukhim et al., 2016)
***Inter-TAD loss- or gain-of-function alterations***		
*IDH locus*	Gliomas	Mutations in the *IDH* gene results in accumulation of 2-hydroxyglutarate, which subsequently represses TET proteins. This causes hyper-methylation of CpG sites and increased methylation of CTCF sites affecting CTCF binding and the respective TAD boundaries. New interactions are consequently established between the oncogene *PDGFRA* with constitutive enhancers, which are normally located outside its normal TAD (Flavahan et al., 2016)
*FMR1 locus*	Fragile X syndrome (FXS)	The CGG triplet repeat (short tandem repeat or STRs) within the *FMR1* gene expands in an erratic way and the *FMR1* locus boundary is disrupted due to inability of CTCF to bound, caused by the abnormal DNA methylation levels. *FMR1* is silenced as the boundary is disrupted, because of the separation from its associated regulatory elements, which are now located in another TAD (Anania and Lupiáñez, 2020).
***Neo-TADs***		
*Kcnj2 and Sox9 loci*	Limb malformation	A neo-TAD where *Kcnj2* interacts with the Sox9 regulatory region resulted in overexpression of *Kcnj2* (Franke et al., 2016)
*IGF2 locus*	Cancer	Due to duplications of neighboring TADs, the new TAD incorporates the *IGF2* gene and a lineage-specific super enhancer, resulting in oncogenic locus mis-regulation (Weischenfeldt et al., 2017)

Interestingly, in multiple myeloma 30% of the breakpoints are located at, or close to, TAD boundaries. The number of TADs is increased by 25% and they are smaller when compared to normal B cells, indicating that genomic rearrangements and translocations are driving forces in chromatin topology and creating new TADs (Wu et al., 2017). The smaller size of TADs in cancer cells when compared to their healthy controls can also be observed in prostate cancer and therefore seem to be most likely a general phenomenon in cancer cells. In the case of prostate cancer, this smaller size is the consequence of the splitting of one TAD in two, the majority of the TAD boundaries (∼98%) being the same between the prostate cancer cells and the normal ones (Taberlay et al., 2016). In prostate cancer, a deletion on 17p13.1 encompassing the *TP53* tumor suppressor locus leads to the division of a single TAD into two distinct smaller TADs, resulting into new chromatin interactomes of the enhancers, promoters and insulators within the TADs and changing gene expression (Taberlay et al., 2016). Similarly, in mammary epithelial and breast cancer several TADs were divided into multiple sub-TADs but kept the same boundaries, as a result of various genomic alterations (Barutcu et al., 2015). In prostate cancer cells (and probably in most cancers) the size of the TADs (2–4 MB) is smaller compared to normal prostate cells (∼8 MB). These new small-TADs reside within the normal TAD architecture rather than forming new TADs, with the majority of the TAD boundaries (∼98%) to be the same between the prostate cancer cells and the normal ones (Taberlay et al., 2016).

Because of their ability to co-localize in the nucleus and/or their abundance within TAD boundaries, transposable elements (TEs) have been related to genome architecture (Dixon et al., 2012; Cournac et al., 2016). It has been shown that during the evolution of mammalian lineages, activation of retro-transposable elements triggered an increase of CTCF-binding events (Schmidt et al., 2012). As shown by changes in chromatin states, many of the new CTCF sites acted as chromatin insulators, affecting genome architecture and transcription. According to this observation, human T-lymphotropic virus type 1 (*HTLV-1*) translocation introduced an ectopic CTCF-binding site, which could form new loops and induce transcriptional changes in the new locus (Melamed et al., 2018). *HTLV-1* results in chronic inflammation in 10% of infect hosts.

Changes in the interactome and local chromatin architecture have also been associated to single nucleotide polymorphisms (SNPs) causing intra-TAD alterations. Intra-TAD alterations lead to abnormal transcriptional control of the genes inside the TAD, without altering its overall conformation. Many GWAS SNPs have now been connected to putative causative genes in hematopoietic cell types (Javierre et al., 2016; Mumbach et al., 2017). How sequence variations in putative regulatory elements lead to gene expression alterations that drive complex illnesses is largely unknown. On one hand, SNPs could restrict TFs or architectural proteins from interacting with their regulatory elements, leading to lower expression of their associated genes (Table 3; Schoenfelder and Fraser, 2019). SNPs can affect the recruitment of the LDB1 complex to the MYB enhancer, impairing its interaction with the MYB promoter, decreasing its expression and resulting in an increase of HbF expression (Stadhouders et al., 2014). On the other hand, SNPs could result in overexpression of target genes and/or their mis-expression in different cell types (Schoenfelder and Fraser, 2019). Gain or loss of function mutations in regulatory elements such as enhancers (or silencers) can affect the transcription of their cognate gene(s) (Spielmann and Mundlos, 2016; Schoenfelder and Fraser, 2019), provided that there is no other regulatory element compensating that gain or loss (Heinz et al., 2013). Another study attempted to identify the causative gene at GWAS neurological disease loci by linking the SNPs with gene promoters and enhancers (Lu et al., 2020). They concluded that a SNP may only have subtle effects on looped target gene in healthy donors, but plays a more prominent role when the locus gains a disease-specific enhancer in patients. Their results indicated that high-quality Hi-C loops have a unique value in the study of disease genetics (Lu et al., 2020). Other GWAS studies have identified mutations in regulatory elements that could contribute to the Inflammatory bowel disease etiology by altering gene expression (Meddens et al., 2016). Duplications can change the copy number of regulatory elements, resulting in loss- or gain-of-function mutations, similar to the principle of gene dosage alterations occurring in the inter-TAD duplications and translocations (Tables 3, 4; Spielmann et al., 2018).

**TABLE 3:** Summary of intra-TAD alterations (Single nucleotide polymorphisms and gain-of-function alterations), the disease/abnormality they caused and their description.

*Single nucleotide polymorphisms (SNPs)*		
*HSBL1-MYB locus*	Hemoglobinopathies	SNPs affect the recruitment of the LDB1 complex to the *MYB* enhancer, impairing its interaction with the *MYB* promoter. Consequently, decrease of *MYB* expression results in an increase of *HbF* expression (Stadhouders et al., 2014)
*CLEC16A locus*	Autoimmune disease	SNPs in intron 19 of the *CLEC16A* gene have been shown to promote the interaction of the intron with the adjacent *DEXI* gene, resulting to its expression (Davison et al., 2012)
*FTO locus*	Obesity	An intron of the *FTO* gene containing obesity-associated SNPs interacts with the distal *IRX3* gene, and thus controlling its expression (Smemo et al., 2014; Schoenfelder and Fraser, 2019)
*SNCA locus*	Parkinson disease	A common Parkinson disease SNP in a non-coding distal enhancer factor prevents two repressive transcription factors, EMX2 and NKX6-1, from binding to a regulatory element, and thus, resulting in *SNCA* transcriptional upregulation (Soldner et al., 2016)
*Various loci*	Chronic Kidney Disease (CKD)	SNPs in both coding and non-coding regions have been discovered in studies of CKD, and dysregulation of gene expression of the 23 genes identified to be associated with such SNPs is possibly a contributing factor in CKD pathophysiology (Brandt et al., 2018)
***Intra-TAD gain-of-function alterations***		
*SHH locus*	Polydactyly	Point mutations in the Sonic hedgehog (*SHH*) regulatory region *ZRS* result in the ectopic expression of *SHH* at the anterior margin in mouse. Although not formerly demonstrated in this study, these mutations allow the formation of chromatin looping between the *ZRS* region and the *SHH* promoter (Lettice et al., 2003).
*MYC locus*	Lung adenocarcinoma	Amplification of *MYC*-regulating enhancers results in a slightly higher *MYC* expression than in samples without amplification of *MYC* enhancers. The enhancer-amplified samples had a comparable *MYC* expression levels when compared to samples with *MYC* coding area amplification (Zhang et al., 2016; Agrawal et al., 2019)
*IHH locus*	Craniosynostosis and synpolydactyly	Duplications of regulatory elements within the *IHH* locus led to misexpression or overexpression of *IHH* and by this affect the complex regulatory signaling network during digit and skull development respectively (Klopocki et al., 2011)
*CTSB locus*	Keratolytic winter erythema	Overexpression of *CTSB* as a result of enhancer duplications (Ngcungcu et al., 2017)
*Various loci*	Prostate cancer	SNPs, associated with prostate cancer, co-localize/affect regions of active histone modification and transcription factor binding sites. 15 of the 17 identified genes in these loci exhibit a substantial change in expression, suggesting that the genes physically interacting with risk loci are associated to prostate cancer (Du et al., 2016)
*Various loci*	Atherosclerotic disease	294 additional candidate expressed genes for coronary artery disease (CAD) and large artery stroke (LAS) have been identified as potential factors in the pathophysiology of human atherosclerotic disease (Haitjema et al., 2017)
*Various loci*	Inflammatory bowel disease (IBD)	Mutations in DNA regulatory elements (DREs) can contribute to IBD etiology by altering gene expression (Meddens et al., 2016)
*Pitx1 locus*	Liebenberg syndrome	Deletion mutations upstream of the hindlimb expressed *Pitx1* gene result in intra-TAD conformation changes, merging a forelimb and hindlimb *Pitx1* gene enhancer (Kragesteen et al., 2018)
*PITX1 locus*	As previous	Translocation of two enhancers from chromosome 18 upstream of the *PITX1* on chromosome 5 (TAD shuffling), resulted in an increased *PITX1* expression (Spielmann et al., 2012)

**TABLE 4:** Summary of intra-TAD alterations (loss-of-function alterations), the disease/abnormality they caused and their description.

*Intra-TAD loss-of-function alterations*		
*Shh locus*	Preaxial polydactyly (PPD)	In the ZRS, two ETV4/ETV5 binding sites have been discovered. In transgenics, a single ETV binding site is sufficient to suppress ectopic expression; the absence of both sites leads in repressor activity loss and, as a result, in ectopic *Shh* expression in the limb bud (Lettice et al., 2012)
*PAX6 locus*	Aniridia	Point mutations (disruption of binding sites) in enhancers of *PAX6*, *PTF1A* and *TBX5* impair the expression of these genes. While it has not been demonstrated properly, these studies suggest that these point mutations impair the chromatin looping between these enhancers and their associated promoters (Smemo et al., 2012; Bhatia et al., 2013; Weedon et al., 2014)
*PTF1A locus*	Pancreatic agenesis	
*TBX5 locus*	Congenital heart disease	
*SOX9 locus*	Campomelic dysplasia	Sex reversal occurs when the relevant testis enhancer of *SOX9* is deleted, while deletions and point mutations further upstream induce the Pierre-Robin syndrome, which is characterized by cranial skeleton growth defects but normal sexual development (Benko et al., 2011)
*DYNC1I1 locus*	Split-hand/split-foot malformation (SHFM)	Exons 15 and 17 of *DYNC1I1* act as tissue specific limb enhancers of *DLX5/6*. Enhancer deletions in the *DYNC1I1* gene result in down regulation of the *DLX5/6* genes about 1Mb away (Allen et al., 2014; Tayebi et al., 2014)
*ATOH7 locus*	Non-syndromic congenital retinal non-attachment (NCRNA)	A deletion that covers a distal cis regulatory element upstream from *ATOH7* is responsible for NCRNA (Ghiasvand et al., 2011)
*SHH locus*	Holoprosencephaly (HPE)	The loss of function (disruption of binding sites) of *Shh* brain enhancer-2 (SBE2) in the hypothalamus of transgenic mouse embryos was caused by a rare nucleotide variant upstream of *SHH gene* found in an individual with HPE (Jeong et al., 2008)
*MYC locus*	Cleft lip with or without cleft palate (CL/P)	Deletion of a 640-kb non-coding region at 8q24, which contains distal cis-acting enhancers that regulate *Myc* expression in the developing face, causes modest facial morphological changes in mice and, on rare occasions, CL/P (Uslu et al., 2014)
*BCL11A locus*	β-hemoglobinopathies	A common variant in an erythroid enhancer of *BCL11A* is associated with reduced TF binding, modestly diminished BCL11A expression, and elevated HbF (Bauer et al., 2013)

While SNPs could alter the content of specific enhancers, resulting to abnormal expression patterns, mutations in genes encoding TFs or architectural proteins could also have similar results (Schoenfelder and Fraser, 2019). Cohesinopathies and laminopathies, however, are the two groups of structural protein-associated human diseases that receive the most attention. Cohesinopathies are caused by mutations in genes associated with Cohesin complex and/or its regulators (Banerji et al., 2017; Norton and Phillips-Cremins, 2017; Davis et al., 2018; Olley et al., 2018; Krumm and Duan, 2019). CTCF and cohesin associated SNPs have been related to a number of human disorders and developmental defects. The significance and role of genome organizing factors like CTCF and the cohesin complex has been highlighted for a number of diseases. For example, CTCF depletion leads to pathological effects that are quite comparable to heart failure (Rosa-Garrido et al., 2017). Altered interactions and accessibility was shown at a substantial number of enhancer areas and the genes in the surrounding chromosomal areas were implicated in cardiac pathological pathways (Rosa-Garrido et al., 2017). Another example are the laminopathies caused by mutations in nuclear lamins (LMNA) and the lamin B receptor (LBR) genes. Given that LADs organize a large portion of the genome, the nuclear lamina and its components appear to play an important role in genome architecture. Laminopathies are distinct from other disorders in that a variety of disorders may be developed from just different mutations located in the same gene (Worman and Bonne, 2007).

**FIGURE 6:**
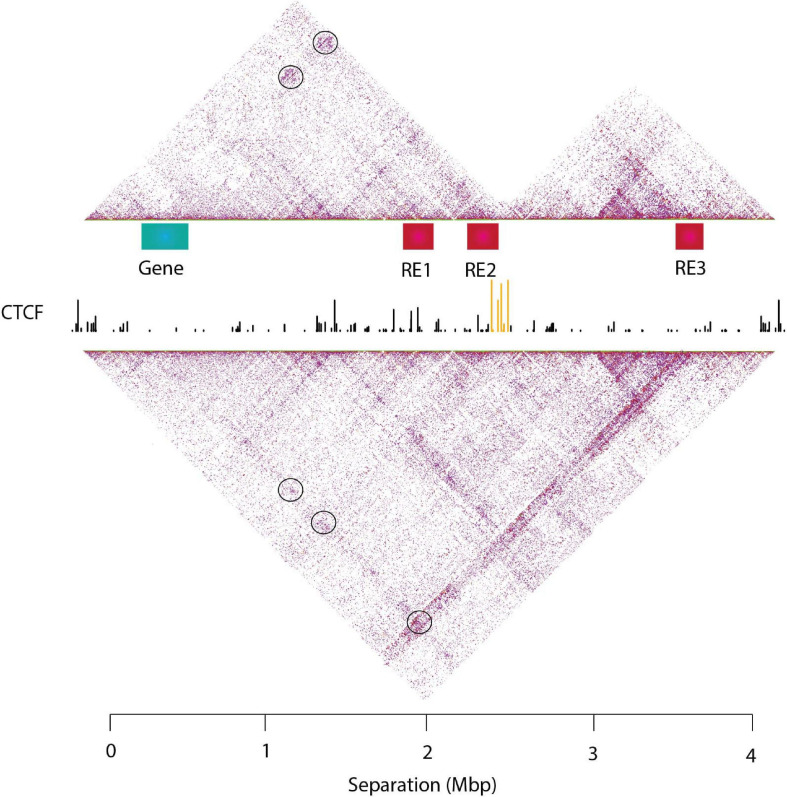
An example of TAD disruption in cancer and rewiring of promoter-enhancer proximity. The upper panel depicts two distinct TADs, the left containing a gene (depicted with a green box) and two regulatory elements (RE1 and RE2 depicted with red boxes), which can be either an enhancer, or a poised enhancer or a silencer. The right TAD contains one regulatory element (RE3) that would be compatible with the gene. In the upper panel, the gene is located in a confined place with RE1 and RE2 (depicted with round black circles) resulting in its normal transcriptional activation (if RE1 and RE2 are enhancers) or its repression (if RE1 and RE2 are poised enhancers or silencers). Mutation or deletion of the CTCF sites (depicted with yellow) located at the boundary between the TADs, results in the reorganization of the TAD topology and fusion of the two TADs into one. Thus, in the bottom panel, the gene is now in close proximity (and interacts frequently) with RE3 (depicted with round black circle), leading to its expression also by the RE3, if RE3 is an enhancer or its downregulation if RE3 is a silencer. The different combinations of REs could have different results in the expression levels of the gene. Since, RE1 and RE2 contacts are diminished, it could lead to less expression by those two enhancers while the expression levels of the gene remain the same. On the other hand, the combination of RE1, RE2, and RE3 could lead to a super-enhancer and higher levels of expression of the gene.

Cancer is a particularly important area of disease where changes in the interactome are important. Alterations in TAD boundaries, which are observed in cancer, can lead to oncogene activation by affecting gene regulation in the flanking TADs via the establishment of new unusual promoter-enhancer interactions (Figure 6). Oncogene activation by TAD disruption and consequent enhancer adoption has been described in leukemia (Gröschel et al., 2014; Hnisz et al., 2016b), neuroblastoma (Peifer et al., 2015), colorectal cancer (Weischenfeldt et al., 2017), medulloblastoma (Northcott et al., 2014), glioma (Flavahan et al., 2016), sarcoma and squamous cancers (Weischenfeldt et al., 2017). Notably, the most prominent alterations in binding sequences at TAD boundaries, are located at CTCF binding motifs (Ji et al., 2016), although it should be noted that many CTCF binding sites are not boundaries. Approximately 11% of 922 deletion cases affect TAD boundaries at the vicinity of a disease-associated gene, resulting in “enhancer adoption” (Swaminathan et al., 2012). A comprehensive analysis among various cancer cell lines, indicated that the formation of neo-TADs, encompassing cancer driver genes, is the result of SV alterations in cancer cells (Dixon et al., 2018). However, whether neo-TAD formation is a recurrent phenomenon in a given cancer cell type needs to be investigated further.

## The Importance of a Refined Identification of Chromatin Conformation and Potential Therapeutic Approaches

An important question is what underlying mechanism protects TAD boundaries from deletions and disruptions? Using machine learning approaches, TAD boundaries were recently categorized based on strength (Gong et al., 2018). Strong TAD boundaries are less frequently lost in cancer, as they act as building blocks of the genome and encompass super-enhancers (Gong et al., 2018). In cancer, strong boundaries are notably safe from SVs and co-duplicated with super-enhancer elements (Gong et al., 2018). These observations and the observations that enhancers lead to aberrant activation of oncogenes due to genetic or epigenetic alterations highlight the importance of the chromatin architecture integrity (Lupiáñez et al., 2015; Flavahan et al., 2016; Hnisz et al., 2016b; Weischenfeldt et al., 2017).

An interesting question is whether mis-regulation of TFs causes the altered 3D chromatin organization or whether the opposite takes place? Intriguingly, studies advocate both options. A gene fusion in prostate cancer, causes the overexpression of oncogenic *ERG* resulting in changes in chromatin organization and territories encompassing genes associated with aggressive prostate cancer (Rickman et al., 2012). This hypothesis may also be true for other TFs whose aberrant expression is involved in many other cancers (Rickman et al., 2012). In contrast, chromosomal inversion and translocation in chromosome 3 at two different breakpoints, tethering the enhancer of *GATA2* in the same TAD as *EVI1*, activate expression of *EVI1* and downregulate *GATA2*, resulting in the development of acute myeloid leukemia (Gröschel et al., 2014).

Thus, is chromatin architecture characteristic of each disease and can we predict the effect of SVs in chromatin organization? A support vector machine classifier (3D-SP) can separate leukemia sub-types based on the information contained in the chromatin architecture and specifically the interactome of the *HOXA* gene cluster in various leukemia cell lines (Rousseau et al., 2014), while a recently developed approach can be used to predict *in silico* the altered 3D conformation resulting from structural variants (Bianco et al., 2018). Hence, the improvement of new chromatin conformation techniques can help to better understand the biological effect of newly discovered structural variants and TAD alterations in the human genome, that are linked to uncharacterized genetic disorders or diseases and to evaluate their role on chromatin architecture and transcriptional control. Interestingly, chromatin conformation capture techniques which employ selection based on oligonucleotides, like T2C (Kolovos et al., 2018) and capture-promoter Hi-C (cpHi-C) (Schoenfelder et al., 2015a), can identify the interactome of those specific fragments. Especially in the cases of where SNPs are heterozygous in these fragments, oligonucleotides designed for the two alleles can discriminate the interactome of the wild type allele compared to the allele containing the SNP.

Targeting chromatin interactions could potentially provide therapeutic approaches (Babu and Fullwood, 2015). Perturbing promoter-enhancer interactions would permit the fine tuning of expression of target genes, in a reversible and specific manner. However, it faces many difficulties that would need to be overcome. CTCF, cohesin and other TFs mediate many different chromatin interactions. Frequently, these TFs are also involved in signaling pathways. Thus, a systemic perturbation of TFs would cause many off-target effects. Moreover, proteins, which mediate chromatin interactions, are often found in the nucleus and are therefore difficult to perturb by antibodies or small molecule inhibitors. Various epigenetic regulators are involved in cancer, but whether they are involved in chromatin organization is poorly understood. Many drugs have been developed for epigenetic regulators but again it has not been examined yet whether they affect chromatin interactions and compartmentalization, although it is likely that many will affect genomic interactions directly by enabling or preventing the binding of TF type protein (e.g., CTCF is DNA methylation sensitive) or indirectly via changes in the transcriptome. Interestingly, a recent study has identified 50 factors that are potentially important for genome organization (Shachar et al., 2015). However, this study applied an siRNA approach, which is known to cause off target effects. To overcome the non-specificity of targeting such proteins, a new tool (CLOuD9) for the precise manipulation of 3D chromatin structure and chromatin looping has been developed by employing the CRISPR/Cas9 approach and establishing stable chromatin loops (Morgan et al., 2017). This approach may be useful in cancer diagnostics, where chromosomal rearrangements interrupt genomic organization and alter gene expression. Thus, screening studies preferably with the use of drugs or CRISPR/Cas9 approach targeting alterations of chromatin conformation structure, could unveil new factors, which mediate chromatin interactions and unveil them as potential new therapeutic targets. More promising would perhaps be the development of genome editing tools to alter the binding sites of TFs or CTCF using Crispr/Cas9 and homing technology to target the appropriate cells (Cruz et al., 2021).

It is clear from the studies above and many others that chromatin conformation plays a key role in cancer. Thus, understanding the modulation of chromatin interactions will unveil the underlying mechanisms of diseases, development and cancer and identify new promising therapeutic targets.

## Conclusion and Future Perspectives

The integrity of the 3D chromatin architecture and the genome interactome is important to ensure proper transcriptional control. Alterations of this topology are often correlated with diseases such as cancer. Since the genome of cancer cells or cells derived from other pathologies are often instable, TAD disruption is observed often, that result in altered gene expression profiles leading to tumorigenesis or other pathology. Hence, mapping the precise location of TAD topology, their boundaries and other structures is an integral part of deciphering the genetic basis of gene expression in cancer and other diseases, and possibly provide new therapeutic targets. Moreover, the recent development of CRISPR-Cas9 technique (Ran et al., 2013) could lead to correcting altered TAD boundaries in patient cells, offering an exciting potential therapeutic strategy. Recently developed high resolution chromatin conformation techniques [e.g., Hi-C (Rao et al., 2014), T2C (Kolovos et al., 2018)] that offer sub-Kbp resolution, could unveil the precise location of TAD boundaries and their detailed features, holding the key to better understand diseases. Finally, we propose a model integrating recent developments in chromatin architecture with the formation of either structural or functional loops, controlling proper transcriptional control. Understanding how these loops are formed and how they evolve is essential to identify new mechanisms triggering pathologies such as cancer and to develop new efficient therapeutic strategies.

However, despite the spectacular recent advances in the field of chromatin architecture and gene regulation, many questions still remain to be answered. Some of those are: is gene activation preceding locus conformation or *vice versa*? What is the underlying mechanism creating TADs and protecting TAD boundaries from deletions and disruptions, e.g., is it continuous loop extrusion? Is 3D conformation accompanying cell lineage decisions? How were regulatory elements generated during evolution? Are there as yet unknown TFs, which contribute to 3D genome structure? How can we efficiently identify these? Deciphering all these questions could further lead to our understanding of the dynamics and forces of chromatin organization to enable all the necessary functions of cells.

## Author Contributions

All authors structured, wrote and proofread the manuscript.

## Conflict of Interest

The authors declare that the research was conducted in the absence of any commercial or financial relationships that could be construed as a potential conflict of interest.

## Publisher’s Note

All claims expressed in this article are solely those of the authors and do not necessarily represent those of their affiliated organizations, or those of the publisher, the editors and the reviewers. Any product that may be evaluated in this article, or claim that may be made by its manufacturer, is not guaranteed or endorsed by the publisher.
